# Decolonizing global health research: experiences from the women in health and their economic, equity and livelihood statuses during emergency preparedness and response (WHEELER) study

**DOI:** 10.3389/fpubh.2025.1578964

**Published:** 2025-03-26

**Authors:** Ahmed Adam, Lisa Lazarus, Bernadette Kina, Robert Lorway, Bilali Mazoya, Michaela Mantel, Marleen Temmerman, Lisa Avery, Evaline Langat

**Affiliations:** ^1^Department of Health Services, Mombasa County Government, Mombasa, Kenya; ^2^Department of Community Health Sciences, Rady Faculty of Health Sciences, University of Manitoba, Winnipeg, MB, Canada; ^3^Institute for Global Public Health, University of Manitoba, Winnipeg, MB, Canada; ^4^Department of Health Services, Kilifi County Government, Kilifi, Kenya; ^5^Center of Excellence in Women and Child Health, Aga Khan University, Nairobi, Kenya; ^6^Department of Obstetrics, Gynecology and Reproductive Sciences, University of Manitoba, Winnipeg, MB, Canada

**Keywords:** decolonization, global health, human-centered design, healthcare workers, partnerships/coalitions, COVID-19, Kenya

## Abstract

Decolonizing global health research involves rethinking power structures and research collaboration to prioritize the voices and experiences of communities that have been historically marginalized. Cross-sectoral and cross-regional partnerships based on reciprocity, trust, and transparency can be facilitated by decolonized research frameworks. To address global health issues in a way that is inclusive, context-specific, and genuinely advantageous to all parties involved, especially communities most impacted by health disparities, the ethics behind this change is imperative. We applied a decolonizing health research approach to the Women in Health and their Economic, Equity, and Livelihood Statuses During Emergency Preparedness and Response (WHEELER) study to explore the connections between gender, health, and economic equity in times of crisis in two counties in Kenya. This paper outlines seven key dimensions that guided the WHEELER study in transforming power dynamics in research, decolonizing research processes, and fostering equitable partnerships. The study employed participatory methodologies, integrating the Equity in Partnership instrument from the Canadian Coalition for Global Health Research (CCGHR) Principles, human-centered design (HCD), and gender-based analysis to ensure inclusivity, gender sensitivity, and active participation. The participatory approach was implemented through the engagement of a Community Research Advisory Group (CRAG) and a Local Advisory Board (LAB). Utilizing mixed methods and community-engaged processes, the study fostered reciprocal growth, learning, and change among local health officials and research teams. Our participatory approach fostered strengthened engagement, promoted shared decision-making, and enhanced the sense of ownership among policy implementers throughout the research process.

## 1 Introduction

Despite growing discourses on “decolonizing global health,” power imbalances between actors from affluent, historically advantaged nations and their peers in the “Global South” continue to see paternalistic approaches to research partnerships, uneven allocations of benefits, and minimal commitments to enhancing local capacity toward research ownership and leadership (1, 2). Even the term “global health” itself entrenches these power hierarchies, enabling certain groups to exploit vulnerabilities under the guise of health interventions (3). Decolonization entails a process of reconstructing institutions and knowledge frameworks while avoiding the cultural and societal consequences of the violence, racism, misogyny, and Western-centrism characteristic of the colonial period (4). This imbalance undermines the principles of mutual respect, shared responsibility, and local empowerment that are fundamental to the Sustainable Development Goal (SDG) calling for revitalizing global partnerships (5).

The recent COVID-19 pandemic is a further reminder of the lingering impacts of colonial and neocolonial dynamics on global health (6). Prioritization of resource allocation to the “Global North” for research funding (7) and vaccine distribution (8) to policy development and execution (9) exacerbated already existing disparities between the Global North and South. The COVID-19 pandemic brought to light how gender norms, caregiving duties, and job segregation imposed extra physical and emotional strain on women (10). In addition to short-term fixes like financing and distribution assistance, addressing these disparities calls for systemic change, including funding for healthcare infrastructure, fair research cooperation, and legislative frameworks that give health justice a top priority in pandemics to come (11).

### 1.1 The shifting landscape of global health partnerships

In recent years, the way research partnerships are conceptualized and carried out has undergone a paradigm shift in the field of global health (12). Power dynamics in these partnerships have come under scrutiny because of the expanding conversation around decolonization and localization (8). This change reflects a wider understanding that conventional Global North-South collaboration models frequently reinforce inequality and fall short of meeting local needs. High-income nations continue to dominate global health agendas. The colonial legacy of global health continues to be seen, with most global health centers located in high-income countries (HICs) playing a disproportionate role at conferences and as senior authors in global health journals (13). In a comprehensive bibliometric analysis of scientific articles published between January 2014 and June 2016 in prominent medicine and global health journals, over one-quarter (28.8%) did not list a local author (14). A systematic review focused specifically on international collaboration in health research in Africa found that while < 15% of publications did not have a local co-author, local authors were less likely to hold first or senior-author positions (15). Assessing the reasons behind the low publication rates of authors from the Global South has often posed challenges. Beyond the crucial drive to publish to gain visibility, having work published in high-impact journals is vital for academic promotion and career progression among all scientists globally (16).

Restrictive visa policies and a lack of prompt provisions by authorities make authors from Global South lose out on important professional opportunities to attend international conferences. Many people experience lengthy processing times, high rejection rates, and financial burdens related to visa applications even though they have been invited to contribute or present, perpetuating worldwide disparities in academic representation (17, 18). More inclusive visa regulations and institutional support are needed to address these systemic issues and promote fairer participation in international academic discourse.

Decolonizing global health and aiming for a future in which researchers form more fair and equitable collaborations are ongoing initiatives (19). To address these inequities, we must act by developing and adopting approaches to research partnerships that work to actively and explicitly confront power hierarchies. Prior efforts to provide guidance and frameworks for addressing hierarchies and decolonizing partnerships have been instrumental in shaping our approach. Foundational works on participatory research methods (20) and critical theories on decolonization (21, 22) have laid the groundwork for rethinking traditional power structures in global health research.

Established frameworks such as Participatory Action Research [PAR; (23)], Community-Based Participatory Research [CBPR; (24)], and decolonizing methodologies (25) prioritize equity, co-creation, and shared power in global health research. By centering community leadership, integrating Indigenous and local knowledge systems, and fostering mutually beneficial relationships, these approaches challenge extractive research practices. Guiding principles from frameworks like the Decolonizing Global Health Movement, the Equity in Partnership principles of the Canadian Coalition for Global Health Research [CCGHR; (26)] anchor this article's perspective, demonstrating how the Women in Health and their Economic, Equity, and Livelihood Statuses During Emergency Preparedness and Response (WHEELER) study's seven dimensions can advance more equitable global health research.

Building on these frameworks, we present seven key dimensions that we found were particularly effective in transforming power dynamics and fostering equitable partnerships within our research partnership. By situating our work within the broader discourse on decolonizing research, we offer our experience as evidence for academics and organizations seeking to co-create knowledge and build meaningful, equitable partnerships. Through this, we aim to demonstrate how collaborative research can act as a catalyst for social justice, rather than perpetuating existing structural inequities.

## 2 Methodological approach

The WHEELER study followed a participatory sequential mixed methods approach. The quantitative method included a survey with a total of 2,526 participants (1,251 from Mombasa and 1,275 from Kilifi). The qualitative interviews consisted of 70 in-depth interviews with paid and unpaid frontline health care providers (HCPs) and 12 key informant interviews with health care managers. For the quantitative survey, participants were chosen at random, with every third HCP invited to participate, however at smaller facilities, all HCPs were included due to the few health care providers in these facilities. For the qualitative study, participants from the survey who had consented to be contacted in the follow up study were invited to participate. Since more than half of the survey participants agreed to be contacted, we purposively selected the participants to ensure a diverse representation of the health care providers.

CCGHR Principles (26) provided a foundational framework for promoting equity, mutual respect, and ethical collaboration throughout the research. A human-centered design [HCD; (27)] participatory approach was used. HCD facilitated an organized approach to idea generation with the community, as well as the development of new solutions based on people's actual needs. The study's analysis was guided by gender-based analysis plus [GBA+; (28)], which allowed for the assessment of access challenges, power imbalances, and differential impacts of COVID-19 on male and female, paid and unpaid healthcare providers.

### 2.1 Ethics

Our study received ethical approval from Institutional Scientific and Ethics Review Committee, Aga Khan University, Kenya (ref: 2022/ISERC_111 V2); National Commission for Science, Technology and Innovation, Kenya (ref: NACOSTI/P/23/23038); and University of Manitoba (UM) Bannatyne Research Ethics Board (ref: HS25777 (H2022: 382). Administrative approval from the two counties was also obtained.

All participants provided informed consent before participation, including a clear explanation of the study's purpose, procedures, potential risks, and benefits. Participants were assured of their confidentiality and the right to withdraw from the study at any time without penalty. Data were anonymized and securely stored to ensure privacy and compliance with ethical standards.

## 3 The wheeler collaborative approach to decolonizing global health

This paper highlights seven dimensions that guided the WHEELER study's efforts to shift power dynamics in research, decolonize research processes, and build equitable collaborations. These dimensions closely align with and respond to key domains highlighted by Faure et al.'s scoping review on equity in international health collaborations (29). We highlight our approaches within each dimension.

### 3.1 Re-balancing power dynamics through collaborative partnerships

Decolonizing global health research requires building a partnership structure that is representative of the countries and communities involved in the research (30). It also involves shifting power by ensuring that local researchers and communities lead the shaping of research agendas, methods, and outcomes. Active local researchers and community involvement in all phases of the research process, from design to dissemination, further aims to transition from extractive research models to co-creative, participatory ones. The Aga Khan University (AKU) in East Africa, the County Government Department of Health of Mombasa and Kilifi in Kenya, and the University of Manitoba (UM) in Canada, partnered to co-create the Women in Health and their Economic, Equity, and Livelihood Statuses During Emergency Preparedness and Response (WHEELER) study, funded by the International Development Research Center [IDRC; (31)].

The study implemented an approach that prioritizes equitable partnerships between Kenyan and Canadian organizations. Two representatives from the County Governments were included as principal investigators, ensuring that leadership extended beyond academia to include health policy decision-makers—a model supported by the funder. Each partner played a vital role: AKU led knowledge mobilization, while UM facilitated two-way capacity building, while the county governments facilitated the contextualization of the study processes and findings as well as guided the formation of the community research advisory group (CRAG) and local advisory board (LAB).

The CRAG and LAB were established to promote engagement, shared decision-making, and ownership throughout the research process. The CRAG included six healthcare providers of different cadres, including three community health volunteers/promoters, while the LAB was comprised of six Department of Health officials from the county health management team (CHMT), medical professionals, and other key stakeholders that are policymakers. Each county had its own CRAG and LAB teams, which were engaged as community representatives at all project stages to provide critical insights into understanding and making sense of project findings.

The research team prioritized inclusivity by actively elevating women academics and junior team members, ensuring that their voices were centered during decision-making processes. This commitment was reflected in the composition of the team, which included three female research assistants and one female master's student. To foster mutual respect and dismantle traditional power structures, the team deliberately avoided using titles, such as “Doctor” or “Professor,” and relied on first names instead. Workshops and project activities created rare opportunities for community members and government representatives to engage in meaningful dialogue, breaking down hierarchical barriers and ensuring that both the researchers and communities were heard. By promoting collaboration with local governments, diverse research teams, and community stakeholders, WHEELER strived to produce research that is not only academically rigorous but also locally relevant and sustainable beyond the project's duration.

### 3.2 Capacity building and reciprocal growth, learning, and change

Investing in infrastructure, leadership, and capacity building in the Global South is necessary to decolonize health research and give local researchers the tools and opportunities to direct research (32). The CRAG and LAB were empowered and engaged at all project stages. Capacity-building efforts prioritized junior researchers and research associates, with a particular focus on supporting women in these roles. WHEELER invested in two resource centers in both Mombasa and Kilifi. The centers empowered local researchers by fostering sustainable and contextually relevant advancements in knowledge and innovation. An online course by the University of Washington on fundamentals of implementation science (33) was sponsored through the study which helped to provide a toolkit to translate study findings to 40 participants.

Capacity building was integrated throughout the research cycle, including planning, development of tools, data collection synthesis, analysis and dissemination. Six “sense-making” workshops were held where the key stakeholders including the CRAG, LAB and research team came together to respond to gaps identified by our team during analysis. The quantitative sense-making workshop facilitated the development of qualitative topic guides, and the identification of emerging themes aligned with the study objectives. Additionally, 44 members from the department, which included the health management team at the county and subcounty level, developed skills on gender in research through a 3-day gender workshop. The gender workshop provided valuable insights into the impact of gender on the lives and work of health workers. It also enhanced participants' understanding and ability to effectively integrate gender considerations into WHEELER's research initiatives and recommendations.

The WHEELER study was able to reflect on the many research processes, allowing for course correction and reciprocal progress. This was attributable to the three constant assessments integrated into the research that looked at research quality, equity and partnership, as well as the use of human-centered design as a participatory method.

Capacity building should focus on decreasing the need for ongoing technical skill support and recognizing bidirectional skill sharing between all members of the research team (29). It is crucial to recognize that after the collaborative effort, both the community partner and the capacity builder are expected to have changed ideas (34). These training sessions facilitated knowledge exchange between the research team and the community, providing insights into data and lived experiences. This approach fostered two-way capacity building, allowing participants to share their expertise while gaining new insights from others, thereby integrating local knowledge with external expertise for more equitable and effective solutions.

### 3.3 Valuing diverse ways of knowing

Decolonization involves valuing diverse forms of evidence and ways of knowing (35). The WHEELER project adopted a methodological framework that blends the CCGHR (26) which centers equity-based principles to guide researchers to adopt more equitable forms of global health research and gender-based analysis plus [GBA+; (36)] with human-centered design (HCD) principles (27). These combined methods aimed to ensure that the study was inclusive, gender-sensitive, and participatory. The participatory approach placed a high value on direct collaboration with the research team, community, and government at every stage of the study. HCD ensured that the needs of the key population were met (32, 37). The objective was to create solutions that deeply resonate with the needs of women healthcare providers during the COVID-19 pandemic, highlighting important challenges and possible areas for development

Our varied workshops on research design, analysis, gender and dissemination brought together participants leveraging their diverse perspectives to create comprehensive frameworks for all aspects of our work. Use of mixed methods and GBA+ further allowed our team to better understand how gender and overlapping risks impact providers' physical and mental health, as well as their job performance—evidence that would not be assessable through survey methods alone.

The workshops provided a safe and inclusive space for meaningful collaboration and knowledge exchange evolving into a symbiotic partnership that departs from traditional, hierarchical approaches to research. The continuous evaluation of the research team ensured that our approach remained inclusive and responsive. An adaptive research design provided flexibility in research techniques necessary to adapt to evolving contexts and new insights that emerged during the project.

### 3.4 Expanding dissemination of knowledge

Decolonization promotes making research more accessible to audiences outside of academia and translating research findings into local languages. Our study used a knowledge broker to diversify knowledge dissemination methods, ensuring they aligned with the needs of varied end users. The broker, who was neither from academia nor the health sector but with experience in writing, helped mitigate disciplinary biases and brought a fresh perspective to communication and positionality. Through in-person consultations, 10 randomly selected potential end users of our project findings identified their preferred dissemination methods. For example, research findings were shared using theater and arts-based approaches, making the information more accessible, engaging, and relatable, especially for audiences less familiar with traditional academic presentations.

Additionally, the process itself fostered inclusiveness, as healthcare providers are rarely consulted on how research findings should be shared, which led them to feel valued as they actively engaged in shaping the dissemination strategies. The dissemination targeted HCP in each county. A total of 194 HCP participated in the dissemination forums across Kilifi and Mombasa. Participants felt a sense of connection as their experiences were reflected and valued, fostering ownership and shared learning. Additionally, the method facilitated a deeper understanding of the research and even generated demand, as healthcare providers preferred theater-based dissemination. To extend its reach, the play was uploaded to YouTube (38) and made accessible via digital platforms.

### 3.5 Supportive funding systems

Decolonizing health research calls for creating equitable funding structures where studies consider local community priorities. WHEELER addressed the priorities of both paid and unpaid female health workers, understanding the unique challenges faced and the need for comprehensive solutions. A significant portion of the funding was directed to local organizations and prioritized junior academics.

The study funder, IDRC, required that local institutions serve as the primary partners, ensuring that 80% of the budget was allocated to the Kenyan lead institutions (39). This approach highlights the role of funders in reshaping power dynamics in global health. While many researchers avoid participatory approaches due to cost concerns, they can be economical when guiding interventions that reach large populations. To drive meaningful change, funding systems must challenge entrenched structures in global health research (40).

### 3.6 Respecting existing local structures

To promote fair research practices and address historical power imbalances in global health, it is essential to understand, recognize, and adhere to local review procedures. This recognition fosters trust and collaboration by ensuring that research projects align with regional ethical standards and community expectations. The WHEELER study upheld these principles by securing ethical approval from the Technical University of Mombasa (TUM), obtaining a National Commission for Science, Technology, and Innovation (NACOSTI) license—a country-level research approval in Kenya—and receiving clearances from the County Departments of Health in Mombasa and Kilifi.

Additionally, the study actively engaged county leadership, strengthening local structures and fostering a sense of ownership and shared learning. Respecting ethical research conduct within countries is crucial. Building relationships and valuing local organizations that support research are key to responsible global health practices. Understanding local partnerships and respecting procedural variations across different contexts ensures ethical integrity. Respectful collaborations do not seek to bypass or undermine these processes but rather engage with and follow them.

Adhering to proper research protocols not only enhances ethical compliance but also facilitates policy uptake. When research is conducted through established local frameworks, it promotes ownership, prevents data extraction without benefit to local communities, and ensures meaningful and sustainable impact. The research team participated in local conferences organized by the Counties of Mombasa and Kilifi, respecting local structures and facilitating broader engagement with the community. This approach enabled wider dissemination of the study findings and encouraged active uptake by local stakeholders.

### 3.7 Shared plan for professional growth

A shared knowledge dissemination plan, including publications, was co-developed by the research team to ensure equal opportunities, particularly for junior researchers and research assistants. A publication matrix was established, allowing early-career researchers to lead in conceptualizing and writing as first authors, supported by writing workshops to foster idea exchange and manuscript development.

Budget allocation prioritized junior team members' participation in conferences and training opportunities to enhance networking and learning. Recognizing the gendered nature of academic partnerships, the collaboration emphasized mentoring junior women researchers by mid- and senior-career women academics, strengthening pathways for professional growth. From the outset, transparency was prioritized, with regular meetings to identify and address growth areas.

### 3.8 Barriers and challenges in implementation of the seven steps

Key barriers included balancing academic rigor with local responsiveness, as maintaining methodological integrity while adapting to community needs proved challenging. This required ongoing negotiation and flexibility from all partners. Another challenge has been navigating power dynamics within the partnership, particularly given the historical context of North-South research collaborations. However, the WHEELER study benefited from clear funding structures that ensured equitable resource distribution to local entities. Sustaining local capacity building remained a persistent challenge, requiring long-term investment beyond the research timeline.

Systemic barriers, such as visa denials, further hindered equitable participation in global academic discourse. Despite budgeting for conference participation and providing mentorship, the study's co-PI and authors to abstracts being presented were denied visas to attend a conference in Canada, underscoring how geopolitical constraints can override institutional support. Addressing these systemic issues requires more inclusive visa policies and strengthened institutional mechanisms to ensure equitable access to global academic platforms.

## 4 Conclusion

For global health research to be truly impactful, power dynamics must be redistributed and decolonized through equitable collaborations. Meaningful partnerships require shared leadership, respect for local knowledge, fair resource distribution, and equitable ownership of research outcomes. While this project represents our efforts at a specific point in time toward decolonizing global health research based on the seven dimensions described above, we acknowledge that we have not addressed all aspects of decolonizing global health research. Reflexivity is essential in this process, particularly in examining the structural and funding mechanisms that largely rely on donor support, which can shape research priorities and dynamics. However, working toward equity in research partnerships remains a challenge beyond this study, underscoring the need for continued efforts to reshape global health research structures.

### 4.1 Lessons learned

Developing flexible research protocols allowed for adaptation based on local input which was essential for ensuring relevance and responsiveness. Transparent decision-making processes and regular equity assessments helped to address power imbalances and fostered inclusive participation. Capacity building should be embedded throughout the research process rather than treated as an add-on, which ensured long-term sustainability. Finally, establishing clear communication channels and maintaining regular stakeholder engagement sessions strengthened collaboration, alignment, and trust among all partners.

### 4.2 Next steps toward decolonizing global health research

The experiences and lessons from the WHEELER project highlight promising pathways for advancing equity in global health research. As the field navigates ongoing challenges related to decolonization and localization, initiatives like WHEELER provide practical models for applying these principles effectively.

Looking ahead, there is a pressing need to embed equitable partnership practices more systematically across global health research. This includes developing standardized tools for evaluating partnership equity, incorporating decolonization principles into research funding structures, and establishing platforms to share best practices and insights.

Ultimately, the future of global health research depends on fostering genuinely collaborative ecosystems where knowledge production is a shared effort rooted in local priorities while contributing to global understanding. The WHEELER project illustrates that such an approach is not only feasible but also crucial for addressing complex global health challenges in a fair and sustainable way.

## Data Availability

The raw data supporting the conclusions of this article will be made available by the authors, without undue reservation.
