# Supplementary material for: Decolonizing global health research: experiences from the women in health and their economic, equity and livelihood statuses during emergency preparedness and response (WHEELER) study
Source: Front Public Health. 2025 Mar 26;13:1578964. doi: 10.3389/fpubh.2025.1578964 (PMC11978830; doi:10.3389/fpubh.2025.1578964)
Supplement: Supplementary file 1 [file Table_1.docx]

**Table** **1. Seven-Dimensions applied towards decolonizing global health research in the WHEELER Study**

| **Dimension** | **Description** | **Application in WHEELER Study** |
| --- | --- | --- |
| **1. Re-balancing Power Dynamics through Collaborative Partnerships** | Shifting decision-making power to local researchers and community stakeholders through collaborative partnerships. | Included local researchers and CRAG and LAB members as co-leaders in study design, data collection, and dissemination to ensure local relevance. |
| **2. Capacity Building and Reciprocal Growth, Learning, and Change** | Providing mentorship, training and resources to local researchers to enhance leadership, skills, and independence. | Two-way capacity building activities such as gender training, publication writing workshops and continuous evaluation of research processes and learning exercises |
| **3. Valuing Diverse Ways of Knowing** | Recognizing and incorporating local knowledge systems and lived experiences into research processes. | Used mixed methods of CCGHR, GBA+, and HCD to highlight gendered risks for female healthcare workers; integrated testimonials to understand unique challenges during COVID-19. |
| **4. Expanding Dissemination of Knowledge** | Ensuring findings are accessible to local audiences by using innovative dissemination methods. | Involved a knowledge broker to identify innovative dissemination methods tailored to the end-users’ needs and preferences |
| **5. Supportive Funding Systems** | Advocating for equitable distribution of financial resources in global health research. | Allocated 80% of the project budget to Kenyan-led institutions, addressing classic funding imbalances. |
| **6. Respecting Existing Local Structures** | Aligning research processes with local ethical guidelines and involving community leaders. | Obtained ethical approvals from Kenyan ethics institutions and worked closely with County Departments of Health in Mombasa and Kilifi fostering a sense of ownership |
| **7. Shared Plan for Professional Growth** | Developing equitable publication plans to ensure recognition of all contributors, especially local researchers. | Co-created a publication strategy with shared authorship; prioritized publishing in local journals to reach Kenyan audiences. |
